# Gut microbial diversity in HIV infection post combined antiretroviral therapy: a key target for prevention of cardiovascular disease

**DOI:** 10.1097/COH.0000000000000426

**Published:** 2017-12-06

**Authors:** Mohamed El-Far, Cécile L. Tremblay

**Affiliations:** a Centre de Recherche du Centre Hospitalier de l’Université de Montréal (CRCHUM); b Département de Microbiologie, Infectiologie et Immunologie, Faculté de Médecine, Université de Montréal, Montréal, Québec, Canada

**Keywords:** cardiovascular diseases, HIV, metabolic pathways, microbiota, probiotic

## Abstract

**Purpose of review:**

Although the HIV-infected population is living longer and getting older under current treatment regimens, significant challenges arise for health management as the infection is associated with various premature aging phenotypes, particularly increased incidence of cardiovascular diseases (CVDs). Here we review the current understanding of HIV-related gut dysbiosis in association with CVD and advances in clinical trials aiming to restore gut microbial diversity.

**Recent finding:**

Identification of a unique signature for gut dysbiosis in HIV infection between different cohorts remains challenging. However, low diversity of microbiota combined with the outgrowth of pathogenic bacterial species together with dysregulated metabolic pathways have been linked to compromised gut immunity, bacterial translocation and systemic inflammation, hence higher CVD risk among different cohorts. Data from recent clinical trials aiming to evaluate the tolerability and efficacy of probiotics in treated HIV+ patients are promising and support a significant increase in microbiota diversity and reduction of systemic inflammation. However, the impact of these microbial and immunological corrections on the prevalence of CVD in HIV+ patients remains unclear.

**Summary:**

Positive immunological outcomes following enrichment of the gut microbial diversity have been documented, and further trials are in progress to evaluate the range of patients, with different immunological backgrounds, who might benefit from these treatments.

## INTRODUCTION

In the modern combined antiretroviral therapy (cART) era, vascular diseases remain a leading cause of mortality in HIV infection [1]. Increasing number of observational studies on cohorts of HIV-infected and treated patients, including ours [2], clearly show that acute myocardial infarction and other vascular diseases such as coronary heart diseases are significantly increased among the infected population [3,4,5^▪^]. A recent meta-analysis on 44 cohorts of HIV+ patients showed increased cardiac morbidities in the infected population and further suggested to consider HIV infection, *per se*, as a vascular risk [6^▪▪^]. Significantly, the same meta-analysis of studies done on 334 417 HIV+ individuals from both United States America and Europe demonstrated significant geographical disparities as to the incidence rates of cardiac diseases and mortality. Both HIV+ and HIV− populations in United States of America were found to have a poorer vascular health compared with their counterparts in Europe, a risk that is clearly accentuated by HIV infection. Although a multitude of risk factors may underlie the impact of these geographical disparities on cardiovascular diseases (CVDs), environmental exposure and more precisely diet, is likely to play a detrimental role. Several studies clearly show that dietary intake in a healthier fashion reduces the cardiac risks [7]. However, food metabolism is largely dependent not only on human enzymes produced by liver and gut mucosa but also on diverse microbial communities present in the gastrointestinal tract (GIT) [8]. Meanwhile, this microbial diversity is compromised in HIV infection and is associated with immune dysfunction and chronic inflammation [9^▪^].

In this review, we will discuss the recent knowledge in the comprehension of the HIV-mediated gut dysbiosis (profound change in the balance between the different microbial species) with some focus on dysbiotic biomarkers in association with CVD. We will also discuss the recent advances in the clinical interventions aiming to promote wider microbial diversities in HIV infection and to reduce risks of inflammation and CVD. 

**Box 1:**
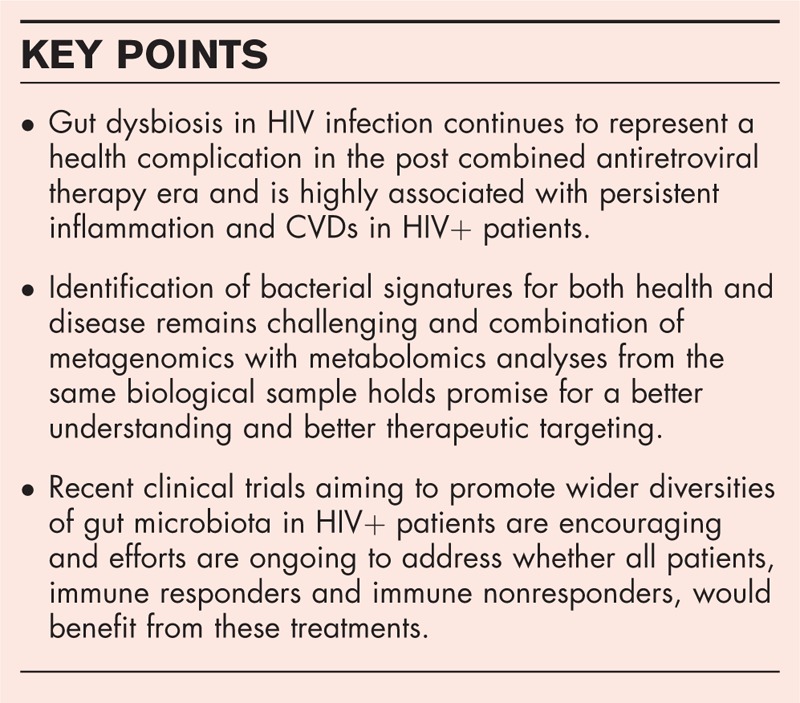
no caption available

## GUT MICROBIOTA AND HEALTH: FROM BIRTH TO DEATH

Human cells are largely outnumbered with resident bacteria (estimated to be >100 trillion bacteria) that live on or within the human body [10,11]. Although it may seem odd, at first glance, to imagine that the human body is submerged with a such tremendous numbers of microbes, body microbiota, especially the commensal gut residents that represent more than 99% of the total body-associated bacteria [12^▪^], are in many ways beneficial for human's health. In addition to its vital role in energy homeostasis, through the impact on host metabolism [13,14], gut microbiota is significantly involved in educating and shaping the two arms of the immune system; both innate and adaptive responses [15]. The importance of these biological processes is reflected by the very early transmission of the mothers’ microbiota to their newborns during delivery [16,17]. Composition of the gut microbiota of newborn babies during the first week of age is similar to that of their mothers and depends on the mode of delivery [18,19]. At the adult stage, composition of the GIT microbiota varies between individuals but remains relatively stable over time within single individuals [20,21]. This composition includes bacteria belonging to different abundant phyla; *Actinobacteria*, *Bacteroidetes*, *Firmicutes* and *Proteobacteria*, but also contains less diverse bacterial phyla such as *Verrucomicrobia*, *Lentisphaerae*, *Synergistetes*, *Planctomycetes*, *Tenericutes* and the *Deinococcus*-*Thermus* group [22]. Accumulating evidence points to this diversity as a key element for a better health as it provides functional redundancy, whereas lower diversity is associated with poorer health, particularly associated with inflammatory diseases [23]. The importance of this diversity is particularly interesting when it comes to aging, frailty and longevity. Recent work on female twins from the TwinsUK cohort showed an inverse correlation between diversity of the gut microbiota and frailty [24^▪▪^]. The *Eubacterium dolichum* (phylum *Firmicutes*) and *Eggerthella lenta* (phylum *Actinobacteria*) species were among the most abundant species that positively associated with frailty, whereas lower *Faecalibacterium prausnitzii* (an anti-inflammatory commensal bacterium [25]) showed an inverse correlation [24^▪▪^]. Significantly, in an effort to identify microbial signatures that differentiate long-living and younger groups on two cohorts of aging centenarian patients from Italy and China, data on gut microbiota showed common greater bacterial community richness [26^▪▪^,^27^^▪▪^]. In the two cohorts, members of the *Clostridium* cluster XIVa (butyrate-producing bacteria), *Ruminococcaceae*, *Akkermansia* and *Christensenellaceae* were enriched in the long-living groups. As all of these bacterial species are potentially beneficial, there is likely a link between longevity and microbiota. However, cause–effect studies in either a clinical setting or in appropriate animal model are still needed to mechanistically confirm these conclusions.

## HIV INFECTION AND DIVERSITY OF GUT MICROBIOTA

To better understand the gut bacterial imbalance under HIV infection and its impact on the overall immune responses, it is first important to recognize the nature of the reference or ‘*normal*’ gut microbial composition under steady state healthy conditions. Meanwhile, it is obvious that defining a healthy microbiome is difficult as it is challenging to define the healthy conditions *per se*. Yet, the Human Microbiome Project (HMP) has defined a set of inclusion and exclusion criteria based on age, overt disease history, use of immunemodulatory drugs or probiotics to define a healthy unbiased cohort of patients that might present minimally perturbed conditions to meet the requirement of the normal microbiome studies [28]. By using these criteria, the HMP reported that the healthy gut microbiota shows a large degree of communities’ diversity and a progression between individuals, from a dominant *Bacteroides* to dominant *Firmicutes* diversities [21]. This diversity is dynamic as interindividual variations are influenced by a variety of environmental, physical, genetic or immunological factors [29]. Therefore, the healthy microbiome might not only be defined by its composition but also by its resilience following insult by either exposure to environmental changes/stresses or following a given host illness [23]. Significantly, an ecological model was proposed by Costello *et al.*[30] suggesting three scenarios for human microbiome assembly; these include development in infants, assembly in the context of invasive pathogenic species and recovery from antibiotics. The last two scenarios typically apply to patients infected with HIV with the exception that this infection remains persistent, even under treatment, and therefore impairment of microbiota diversity is likely to also persist. As HIV targets CD4 T cells, the virus can technically impact all body lymphoid, mucosal and nonlymphoid organs in which these cells can reach and so impacts the associated microbiome. Although true, yet HIV infection is mainly considered as a gut disease as it significantly depletes CD4 T cells from mucosal sites, particularly from gut-associated lymphoid tissues [31]. HIV also induces apoptosis of gut enterocytes, local inflammation (increased TNFα) and promotes dysregulated gut permeability, all of which significantly alters the GIT epithelium structure/function and the overall intestinal immunity [32–35]. In addition, HIV infection depletes mucosal Th17 cells that play a critical role in the antimicrobial defense [36–39]. Together, these alterations are likely to impact the composition of the gut microbial communities leading to dysbiosis. Vujkovic-Cvijin *et al.*[40] showed that gut microbiota from HIV-infected patients is enriched with genera from the *Enterobacteriaceae* family that includes members known to be associated with chronic inflammation such as *Salmonella, Escherichia, Serratia, Shigella* and *Klebsiella* species. Mutlu *et al.*[41] also showed that the lower GIT of HIV-infected patients is enriched with a number of potentially pathogenic bacteria such as *Prevotella* and, in contrast, has poor content of the commensal *Bacteroides*. This was also associated with increased systemic inflammatory cytokines such as IL-6 and TNFα. Significantly, HIV+ elite controllers, a subgroup of patients having the unique capacity to control viremia in the absence of treatment, have a richer gut microbiota compared with progressive patients who depend on cART treatment. Elite controller patients were shown to have enriched genera such as *Succinivibrio*, *Sutterella*, *Rhizobium*, *Delftia*, *Anaero lum* and *Oscillospira* but depleted in *Blautia* and *Anaerostipes*[42^▪▪^].

## COMPOSITION OF GUT MICROBIOTA AND ALTERATION OF METABOLIC PATHWAYS

**FIGURE 1:**
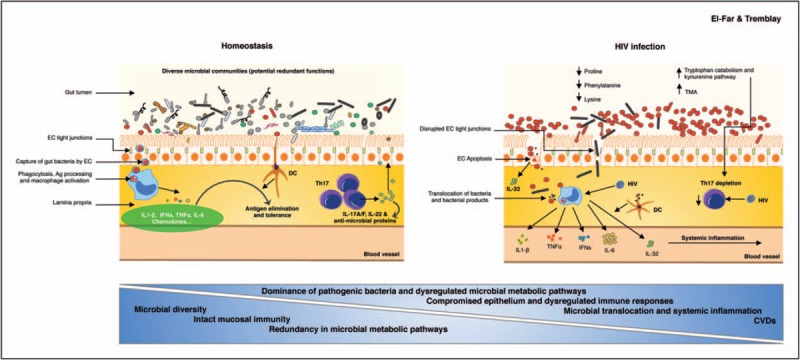
Lower microbial diversity and dysregulated bacterial metabolic pathways accentuate systemic inflammation in HIV infection. Panel (a) under steady state conditions, gut lumen contains a significant diversity of bacterial communities that differ between individuals but remain relatively stable within single individual. Intact gut epithelium together with normal innate responses mediated by macrophages and dendritic cells, as well as with the Th17 normal T-cell functions (production of IL-17A and IL-17F, IL-22 and induction of antimicrobial proteins such as defensins), protects against microbial translocation and further inflammation. Panel (b) under HIV infection, gut epithelium integrity is compromised by epithelial cell death through apoptosis [45] and weakened tight junctions between cells [46] thus leading to enhanced translocation of microbes and microbial products. Together with the HIV-mediated depletion of Th17 cells [37] and enhanced expression of cytokines such as IL-32 [47] that amplifies the inflammatory process, this induces a persistent activation of immune cells and high levels of inflammatory cytokines in circulation. The compromised gut immune response is associated with a decrease in the diversity of microbiota composition and a dysregulated metabolism (lower production of certain amino acids such as proline, phenylalanine and lysine and accelerated catabolism of others such as tryptophan) [44^▪^]. Accelerated tryptophan catabolism leads to increase in the kynurenine pathway, which is involved in the decrease of Th17 cell levels, thus further promoting a biased mucosal immunity. Dysregulated microbial metabolism also leads to increase in the trimethylamine [48^▪^], a precursor for trimethylamine-*N*-oxide, a molecule involved in thrombosis risk [49^▪^]. Ag, antigen; DC, dendritic cells; EC, epithelial cells.

HIV-induced changes in the composition of gut microbial communities is associated with metabolic alteration that, on their turn, would lead to adverse clinical outcomes. Recent data comparing progressor patients with elite controllers, showed that elite controllers have a distinct microbiota metabolic profile that favors fatty acid metabolism, peroxisome proliferator-activated receptors-signaling and lipid biosynthesis protein pathways combined with a decrease in carbohydrate metabolism and secondary bile acid synthesis [42^▪▪^]. Furthermore, other studies showed that progressive patients are enriched with bacterial communities that catabolise the essential amino acid tryptophan through their capacity to produce the rate-limiting enzyme indoleamine 2,3-dioxygenase 1 [40]. The rate of tryptophan catabolism is known to be increased in HIV infection and is associated with disease progression [43]. Increased levels of tryptophan catabolites, particularly 3-hydroxyanthranilic acid is directly involved in the biased balance of Th17 to T_reg_ cells which further contributes to immune suppression, bacterial translocation and systemic inflammation [43]. More recently, Serrano-Villar *et al.*[44^▪^] showed viral-induced quantifiable metabolic changes specific to HIV. Using liquid chromatography coupled with mass spectrometry, this study reported a metabolic deficit in the gut microbiota of HIV-infected patients with impaired capacity to produce three amino acids: proline, phenylalanine and lysine. In contrast, but in agreement with the study of Vujkovic-Cvijin *et al.*[40], there was an accumulation of the tryptophan metabolite 3-hydroxyanthranilate. Significantly, tryptophan catabolism and the kynurenine pathway are inversely correlated with microbiota richness [42^▪▪^] and thus highlighting the importance of the microbial metabolic pathways in the overall immune status as summarized in Fig. 1.

## MICROBIOTA AND METABOLOMICS STUDIES IN HIV AND CARDIOVASCULAR DISEASE

Diet metabolism by gut microbiota produces small-molecule metabolites that interfere with gut physiology. Measuring these metabolites by metabolomic profiling, although not novel, has gained a significant interest over the past decade due to major advances in the high-throughput technologies that permit identification of a large number of metabolites from a single biological sample [50–52]. Using these technologies, Wang *et al.*[53] identified three metabolites of the dietary lipid phosphatidylcholine, choline, trimethylamine-*N*-oxide (TMAO), and betaine, that promote and predict risk for CVD. At the mechanistic level, TMAO alters calcium signaling, cholesterol and bile acid metabolism, fosters activation of inflammatory pathways and promotes foam cell formation, all of which are linked with atherogenesis [54]. TMAO also increases platelet hyper-reactivity, which is associated with cardiometabolic diseases and potential risks of thrombosis [49^▪^]. Of note, TMAO production was shown to vary between men groups based on individual's diversity of the gut microbiota [55^▪^]. Patients with higher *Firmicutes* to *Bacteroidetes* enrichment had higher TMAO production. More recently, Rath *et al.*[48^▪^] established databases for the key genes of the main trimethylamine (a precursor of TMAO) synthesis pathways that permitted the identification of bacterial producing communities, *Clostridium XIVa* strains and *Eubacterium* sp. strain AB3007 among others. Significantly, a meta-analysis on 19 prospective studies showed that blood TMAO and its precursors are associated with elevated risk of major adverse cardiovascular events and a higher all-cause mortality independently of traditional risk factors [56^▪^]. However, a recent study in HIV infection failed to show any association between TMAO levels and platelet-hyperactivity in both treated and untreated patients [57^▪^], although TMAO levels were elevated. Rather, the study showed a significant association between TMAO and sCD14 and a higher ratio of TMAO to its precursors carnitine and betaine in treated patients. The lack of direct association with platelet hyperactivation in this study may in part be explained by the multitude of HIV-associated factors that may interfere with platelet activation. This may also explain earlier reports showing an inverted U-shaped association between TMAO levels and the presence of coronary artery stenosis among HIV-infected men [58^▪^]. In this last study, it was only the middle subpopulation within the second and third TMAO quartiles, compared with the first and fourth quartile, that showed an association with coronary stenosis, which suggests the involvement of other pathways. In this regard, Haissman *et al.*[57^▪^] suggested a role for cART in TMAO metabolism. This observation together with the fact that not all patients with high TMAO levels will experience a cardiac complication limits the role of TMAO as a strong predictor of CVD. However, further studies are needed to dissect the TMAO metabolic pathways to investigate whether there are other compensatory mechanisms that counteract TMAO functions under significantly higher levels of this small molecule. In addition, profound metabolomic studies coupled with microbiota diversity in HIV-infected and treated patients are still needed to uncover other metabolites that may better predict CVD.

## RESILIENCE OF MICROBIOTA ASSEMBLY BY CLINICAL INTERVENTION

Administration of a single or multiple biological components of microorganisms (probiotics) into HIV-infected patients to attain resilience of gut microbiota assembly and hence recovery of important metabolic pathways seems to be clinically feasible. A number of successful clinical trials show a clinical benefit from administrating probiotics. For instance, the Probio-HIV Clinical Trial administered a 1-g packet containing a mixture of different species of bacteria belonging to *Lactobacillus* and *Streptococcus* to cART-treated HIV+ patients twice a day for 48 weeks, leading to a decrease in CD4 T-cell activation, lower levels of sCD14 and Lipopolysaccharide-binding protein (LBP) as well as C-reactive protein (CRP) (a biomarker for CVD risk) [59^▪^]. Significantly, in the ProGut clinical trial (a double-blind study on 32 patients receiving cART but having CD4 counts below 500), daily self-administration of fermented skimmed milk supplemented with different *Lactobacillus* and *Bifidobacterium* subsp. for 8 weeks leads to a significant decrease in CRP, IL-6 and d-dimer, all of which are considered as inflammatory risk markers for CVD [60^▪^]. Similarly, studies on probiotics supplementation with *Saccharomyces boulardii* in HIV-1-infected patients with virologic suppression showed decreased systemic inflammation (lower IL-6) and microbial translocation (lower LBP) [61]. Furthermore, *S. boulardii* treatment significantly decreased bacterial species within the *Clostridiaceae* family, which were correlated with systemic levels of bacterial translocation and inflammation markers at baseline prior to treatment [62^▪^].

Fecal microbial transplantation (FMT) is another clinical intervention to promote gut microbial diversity and healthier metabolic environment through the transfer of the bacterial communities in stool isolated from a healthy donor. This type of transfer although shows significant results in the treatment of *Clostridium difficile* infection (CDI) [63] does not normalize gut microbiota of HIV infected and treated patients [64^▪^]. The microbiota taxa remained restrained with no significant impact on the inflammatory markers monitored in this study. Indeed, in a study to treat CDI, fecal transplantation promoted complete resolution of symptoms in patients with CDI alone but failed in patients with inflammatory bowel diseases (IBD) [65]. As HIV and IBD are both considered as inflammatory gut diseases, the lack of success of FMT treatment in both cases may reflect a common pathway of failure that might be linked to the significant level of intestinal insult and inflammation in these patients. Further studies will likely address these questions. Of interest, pilot clinical trials (PROOV IT I and PROOV IT II) to study the impact of probiotics on the gut microbiota in HIV-infected, cART-naïve or cART-treated with poor CD4 recovery, are currently in progress and results from these studies will likely guide future avenues for microbiota studies in patients with marked gut inflammation [66].

## CONCLUSION

Gut microbiota is now acknowledged as a potential target for biotherapies to dampen inflammation and risks to develop CVD. However, due to the large diversities between individuals in terms of microbiotal composition, it remains challenging to identify a signature for the optimal microbial make-up that might protect against these serious health complications. Coupling the microbiota metagenomics with high-throughput metabolomics technologies in the same study represents an opportunity to understand not only the individual's diversities but also the functional microbiota in terms of microbe–microbe and microbe–host interactions. Finally, whether or not a single regimen of microbial supplementation or a combined therapy targeting a particular metabolic pathway would be necessary to promote recovery of a healthier gut and lower CVD risk is not yet clear and still waiting for larger clinical trials on different populations that should consider geographical disparities.

## Acknowledgements

Thanks to Ms Sonia Deschênes for her careful reading of the article.

### Financial support and sponsorship

M.E.-F. and C.L.T.'s research is funded through National Institutes of Health in the United States, NIH (1R01AG054324-01) and the Canadian Institutes of Health Research, CIHR (PJT 148482).

### Conflicts of interest

There are no conflicts of interest.

## REFERENCES AND RECOMMENDED READING

Papers of particular interest, published within the annual period of review, have been highlighted as:

▪ of special interest
▪▪ of outstanding interest
